# Feed intake, digestibility and energy partitioning in beef cattle fed diets with cassava pulp instead of rice straw

**DOI:** 10.5713/ajas.17.0759

**Published:** 2018-03-13

**Authors:** Kanokwan Kongphitee, Kritapon Sommart, Thamrongsak Phonbumrung, Thidarat Gunha, Tomoyuki Suzuki

**Affiliations:** 1 Department of Animal Science, Faculty of Agriculture, Khon Kaen University, Khon Kaen 40002, Thailand; 2 Bureau of Animal Nutrition Development, Department of Livestock Development, Ratchathewi, Bangkok 10400, Thailand; 3 Animal Production and Grassland Division, Japan International Research Center for Agricultural Sciences, Tsukuba, Ibaraki 305-8686, Japan

**Keywords:** *Bos indicus*, Intake, Digestibility, Rumen Microbes, Energy Requirement

## Abstract

**Objective:**

This study was conducted to assess the effects of replacing rice straw with different proportions of cassava pulp on growth performance, feed intake, digestibility, rumen microbial population, energy partitioning and efficiency of metabolizable energy utilization in beef cattle.

**Methods:**

Eighteen yearling Thai native beef cattle (*Bos indicus*) with an average initial body weight (BW) of 98.3±12.8 kg were allocated to one of three dietary treatments and fed *ad libitum* for 149 days in a randomized complete block design. Three dietary treatments using different proportions of cassava pulp (100, 300, and 500 g/kg dry matter basis) instead of rice straw as a base in a fermented total mixed ration were applied. Animals were placed in a metabolic pen equipped with a ventilated head box respiration system to determine total digestibility and energy balance.

**Results:**

The average daily weight gain, digestible intake and apparent digestibility of dry matter, organic matter and non-fiber carbohydrate, total protozoa, energy intake, energy retention and energy efficiency increased linearly (p<0.05) with an increasing proportion of cassava pulp in the diet, whereas the three main types of fibrolytic bacteria and energy excretion in the urine (p<0.05) decreased. The metabolizable energy requirement for the maintenance of yearling Thai native cattle, determined by a linear regression analysis, was 399 kJ/kg BW^0.75^, with an efficiency of metabolizable energy utilization for growth of 0.86.

**Conclusion:**

Our results demonstrated that increasing the proportion of cassava pulp up to 500 g/kg of dry matter as a base in a fermented total mixed ration is an effective strategy for improving productivity in zebu cattle.

## INTRODUCTION

Beef cattle feeding systems that do not meet the energy requirements of the animals may result in the failure of livestock to meet performance expectations [1,2]. The major reasons for low productivity in tropical developing countries are the genetic potential of the animals, the available feeds and the feeding systems, which depend on rice straw or other low-quality crop by-products and thus limit feed intake and digestion, productivity and environmental sustainability [3–6].

Currently, there is an increasing surplus of cassava (*Manihot esculenta* Crantz) pulp that is available year round for animal feed because Thai cassava starch is an industrial by-product. Cassava contains abundant digestible starch and fiber, and its nutritive value makes it suitable as an alternative energy feed source to rice straw [7–9]. The use of high-energy-dense diets, which typically contain high levels of non-fiber carbohydrate (NFC) to improve digestibility, reduces enteric methane intensity in beef cattle because the degradation of NFC in the rumen affects the activity of methanogenic archaea and other microbes leading to suppressed methane production.

Rumen microorganisms play a major role in providing some of the energy requirements of the host animal by transforming the organic compounds in feed to yield usable energy. Energy requirements are a function of energetic efficiency; therefore, energetic efficiency must be known to determine the energy supply needed from a diet to meet production targets [10–12]. Our previous studies [5–6,13,14] have consistently found that energy required for maintenance during the fattening phase of zebu (*Bos indicus*) beef cattle is approximately 15% less than that for European cattle. A recent meta-analysis indicated that *Bos indicus* used metabolizable energy (ME) more efficiently for maintenance [15,16]. However, to our knowledge, limited information is available on energy utilization by yearling Thai native beef cattle. The objective of this study was to investigate the effects of diets with differing proportions of cassava pulp and rice straw as a base in fermented total mixed rations (FTMRs). We assessed growth performance, feed intake, digestibility, rumen microbial populations, energy partitioning and the efficiency of ME utilization in yearling Thai native beef cattle.

## MATERIALS AND METHODS

The experiment was conducted at the research farm of the Faculty of Agriculture, Khon Kaen University in Khon Kaen Province, Thailand (latitude 16.48°N, longitude 102.82°E). The mean temperature and humidity during the experiments were 26.6°C±4.2°C and 84.9%±5.7%, respectively. All of the procedures involving live animals were approved by the Animal Ethics Committee of Khon Kaen University (Reference No. 0514.1.12.2/98).

### Animals and housing

Eighteen yearling Thai native beef cattle with an average age of 15.02±4.39 months and initial body weight (BW) of 98.3± 12.8 kg were used in the experiment. The animals were selected from a herd held at the Udon Thani Animal Nutrition Development Station, Udon Thani Province and were transported to Khon Kaen University. Upon arrival, the animals were housed individually in adjacent holding pens (2.5×4.5 m) with free access to diet and drinking water throughout the experiment. Each animal was treated for intestinal and external parasites (1 mL/50 kg BW; Ivermectin, L.B.S. Laboratory, Bangkok, Thailand) and given an intramuscular injection of A, D_3_, E vitamins to improve health (3 mL/head; Phoenix Pharmaceuticals N.V., Antwerp, Belgium).

### Experimental design, dietary treatments and silage preparation

The experimental animals underwent an adaptation period of 18 days before the 149-day feeding period. The individual animals were considered the experimental units and were each assigned to one of six blocks (replicates) according to sex and initial BW using a randomized complete block design. Within each block, the animals were randomly assigned to one of three dietary treatments.

The three dietary treatments included the substitution of rice straw with cassava pulp in the following proportions: 100, 300, and 500 g/kg dry matter (DM) basis (Table 1). The diets were formulated to contain 9.6 to 12.4 MJ ME/kg DM and iso-nitrogenous compounds (approximately 10% crude protein [CP]) to meet or exceed the nutrient requirements of Thai native cattle weighing 100 to 250 kg and with a target average daily weight gain of 500 g/d, according to the guidelines of the Working Committee of Thai Feeding Standards for Ruminants (WTSR) [4]. The ingredients and chemical compositions of the experimental diets are given in Table 1. The diets were offered *ad libitum* to all cattle twice per day at 08:30 and 16:30 as FTMRs [17].

A total of 15 bales of FTMR (5 bales per treatment) were prepared monthly using a horizontal mixer with a 1,000-kg capacity (Pak Thong Chai Pasusat, Nakhon Ratchasima, Thailand). Approximately 350 kg of each batch of the treatment mixtures was mixed and then packed and ensiled in sterilized polyethylene bags (220-cm high×150-cm long×0.14-mm thick, Sahavanit Industry Co., Ltd., Bangkok, Thailand), vacuum compressed (Imarflex 1800 W model VC–910, Imarflex Industrial Co., Ltd., Bangkok, Thailand) and covered with a thread bag lid. The bags were stored at outdoor ambient temperatures (approximately 27°C to 35°C) for at least 7 days. After each bale was opened, approximately 2 kg were taken from the outside and inside of the upper, center and bottom portions of the bale and mixed well before determining the quality of the FTMRs.

### Feed intake and digestibility

Feed offered and feed refused were weighed and recorded for each animal daily. The daily feed intake per individual was calculated as the difference between the feed offered and the feed refused throughout the feeding period.

Animals in each block were maintained in individual pens for 3 days before being moved in random sequence to a metabolism cage equipped with head boxes for 6 consecutive days for feces and urine total collections and measurements of gaseous exchange. The excreted feces from each animal were immediately collected into pans placed behind the animal and weighed daily. Total urine volume was collected through a funnel into 5-L plastic buckets containing 6 N HCl to maintain the pH at <3.0. The amounts of feces and urine voided were recorded, and daily samples of 10% total excretion were collected and stored at 4°C. Immediately after completion of the metabolic-data collection period, 1-kg daily aliquot samples of the feed offered, feed refused and feces produced, as well as 500 mL of excreted urine, were thoroughly mixed and stored at −20°C until chemical analysis.

The animals were weighed at 07:30 at the beginning of the experiment and then monthly throughout the experiment to determine average daily weight gain.

### Animal calorimetry and energy utilization measurements

Respiratory gas exchange measurements were conducted during the last three days of the metabolic collection period at 4-min intervals for 23.5 h/d (from 08:30 h to 08:00 h of the following day). The animal calorimetry system was used to measure oxygen consumption and carbon dioxide and methane production according to the methods of Suzuki et al [18]. The system consisted of 3 metabolic crates, each with a head box system and flow meter (Model NFHY–R–O–U, Nippon Flow Cell, Tokyo, Japan) that was used to record the flow rate (147.4 ±21.9 L/min; mean±standard deviation) and the total volume of air flowing through the system. The oxygen concentration was analyzed using a dual-chamber paramagnetic oxygen analyzer (4100 Gas Purity Analyser, Servomex Group, East Sussex, UK). Carbon dioxide and methane were measured using an infrared gas analyzer (IR200 Infrared Gas Analyzer, Yokogawa Electric Co., Tokyo, Japan). The gas analyzers were calibrated daily against certified gases (Takachiho Chemical Industrial Co., Tokyo, Japan); reference gases consisted of two concentrations of oxygen (19.0% and 20.6%), 1.89% carbon dioxide, and 1,960 ppm of methane.

Metabolizable energy intake (MEI) was calculated by subtracting the urine and methane energy outputs from the digestible energy (DE) intake. Heat production (HP) was estimated from the measurements of oxygen consumption, carbon dioxide and methane production, and urinary nitrogen production using the Brouwer [19] method. The average of the antilog of the intercept of the linear regression between the log of HP and MEI was used to estimate the net energy requirement for maintenance (NE_m_) and the efficiency of ME utilization for maintenance (*k*_m_) following the method of Lofgreen and Garrett [10]. Energy retained (ER) was calculated by subtracting the heat production from MEI, and the linear regression of ER on MEI produced the slope assumed to be the efficiency of energy utilization for growth (*k*_g_) and estimate ME requirement for maintenance (ME_m_) using ARC [11]. An alternative method, the intercept divided by the slope of the regression of ER on MEI above maintenance was used to compute the adjustment of MEI for the ME_m_ requirement [15,16].

### Chemical analyses

Feed samples were collected weekly to determine the DM, and samples from four consecutive weeks were pooled for chemical analyses. The DM contents of feed offered, feed refused and feces were determined by oven drying at 105°C to a constant weight. Corresponding subsamples (900 g wet weight) were dried in an oven at 55°C to a constant weight and then ground in a sample mill and passed through a 1-mm screen prior to chemical analysis.

The feed offered, feed refused and feces produced were analyzed using the AOAC [20] procedures for DM, ash, ether extraction (EE) and CP determination (methods 967.03, 942.05, 920.39, and 984.13, respectively). Neutral detergent fiber (NDF) (assayed with a heat-stable amylase and expressed inclusive of residual ash) and acid detergent fiber (ADF) (expressed inclusive of residual ash) were analyzed with a fiber analyzer (ANKOM 200/220, ANKOM Technology, Macedon, NY, USA), according to the method of Van Soest et al [21]. The acid detergent lignin (ADL) of feed offered was analyzed by the solubilization of cellulose with sulfuric acid according to the method of Galyean [22]. NFC content was estimated according to the equation NFC (g/kg DM) = 1,000–(CP+ NDF+EE+ash). The nitrogen content of the urine was determined following the AOAC [20] (method 984.13). The gross energy (GE) contents of the feed offered, feed refused, and feces and urine voided were determined using a bomb calorimeter (IKA C2000 Basic, IKA-Werke, Staufen, Germany).

To determine the fermentation quality of the FTMRs, a 20-g fresh sample of each FTMR was homogenized with 180 mL of sterilized distilled water and stored overnight at 4°C. The macerated sample was then filtered through filter paper (Whatman Grade No. 4 Filter Paper, Buckinghamshire, UK), and the acidity of the filtrate was immediately determined using an electrode pH meter. Supernatants were prepared for analysis of ammonia nitrogen (NH_3_-N), lactic acid and volatile fatty acids (VFAs). The concentration of NH_3_-N was analyzed according to the method described by Fawcett and Scott [23] using a spectrophotometer (UV/VIS Spectrometer, PG Instruments, London, UK), and the levels of lactic acid and VFAs were analyzed according to the method described by Porter and Murray [24] by using a gas chromatograph (GC2014, Shimadzu, Tokyo, Japan) equipped with a flame ionization detector and a 25-m×0.53-mm capillary column (BPX5, SGE Analytical Science, Victoria, Australia).

### Evaluation of rumen microbial populations by real-time polymerase chain reaction

Rumen fluid was collected 3 h after feeding for genomic DNA extraction using a Fast DNA Kit (MP Biomedicals, Cleveland, OH, USA). The DNA concentration was quantified using a spectrophotometer at 260 nm and purified (Nucleo Spin Gel and PCR Clean-up, Macherey-Nagel, Düren, Germany). The CFX96 Touch real-time polymerase chain reaction (PCR) system (Bio-Rad Laboratory, Hercules, CA, USA) was used to determine the relative abundances of total protozoa, total bacteria, *Fibrobacter succinogenes* (*F. succinogenes*), *Ruminococcus flavefaciens* (*R. flavefaciens*), *Ruminococcus albus* (*R. albus*), total methanogens and rumen cluster C archaea populations in the rumen using published primers [25–29]. The amplification reaction contained 10 μL of KAPA SYBR Green master mix (Kapa Biosystems, Woburn, MA, USA), 7 μL of water, 2 μL of DNA template and 0.5 μL of 500 nmol of each primer. PCR amplification was performed following the assay conditions as described in Kaewpila [30].

### Statistical analysis

All of the data were subjected to analysis of variance using the general linear models procedure of SAS 6.12 (SAS Inst. Inc., Cary, NC, USA). The model included terms for treatment (df = 2) and block (df = 5) according to the following equation: Y_ij_ = μ+τ_i_+β_j_+ɛ_ij_, where Y_ij_ is the dependent variable; μ is the overall mean; τ_i_ is the fixed effect of dietary treatment; β_j_ is the fixed effect of block; and ɛ_ij_ is the residual error. Dietary treatment effect and linear and quadratic contrasts were evaluated using contrast statements in SAS to determine the effect of replacing rice straw with cassava pulp in the diets.

## RESULTS

### Animal performance and rumen microbial populations

The growth performance, DM intake, nutrient intake and digestibility results are shown in Table 2. An increase in the proportion of cassava pulp in the diet was associated with a linear increase (p<0.05) in average daily gain and the digestible DM, organic matter (OM), and NFC intakes of the animals. In contrast, the intake of ADF decreased linearly with increasing proportion of cassava pulp (p<0.05), whereas the CP and EE intakes were not significantly different (p>0.05) among the treatments. The apparent digestibility of DM, OM, NDF, and NFC were significantly higher (p<0.01) with an increasing proportion of cassava pulp in the diet, whereas apparent digestibility of CP, EE, and ADF were not affected (p>0.05) by diet treatment.

Rumen microbial population levels are presented in Table 3. The total protozoa population increased linearly with an increasing proportion of cassava pulp in the diet (p<0.05), whereas the populations of *F. succinogenes*, *R. flavefaciens*, and *R. albus* decreased linearly with increasing cassava-pulp proportion (p<0.05). Diet treatment did not influence (p>0.05) ruminal pH, the total bacterial population, total methanogens, or rumen cluster C archaea in the rumen.

### Energy partitioning

The energy partitioning results are shown in Table 3. As the proportion of cassava pulp increased, there was a significant linear decrease in energy loss in urine (p<0.01). Enteric methane energy loss and heat production were not affected (p>0.05) by the proportion of cassava pulp in the diet. There were linear increases (p<0.05) in daily energy intake, retention and efficiency with an increasing proportion of cassava pulp in the diet (Table 3).

**Figure 1:**
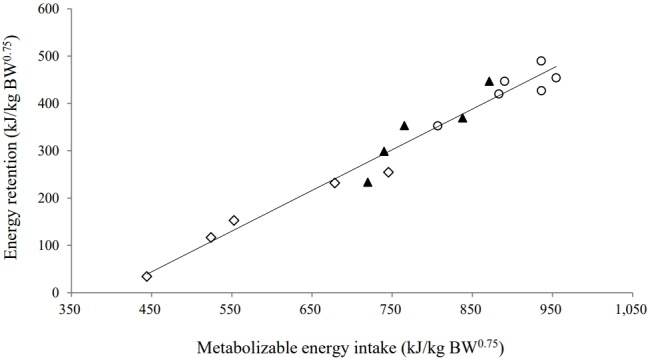
Linear regression of energy retention (ER, kJ/kg BW^0.75^) against metabolizable energy intake (MEI, kJ/kg BW^0.75^) of yearling Thai native beef cattle fed fermented total mixed ration diets with different proportions [100 (⋄), 300 (▲), and 500 (○) g/kg dry matter basis] of cassava pulp instead of rice straw. Equation: ER = (0.860_(SE=0.05)_×MEI)–342.7_(SE=35.96)_ (*r* ^2^ = 0.96; p<0.001; residual standard deviation = 6.9).

**Figure 2:**
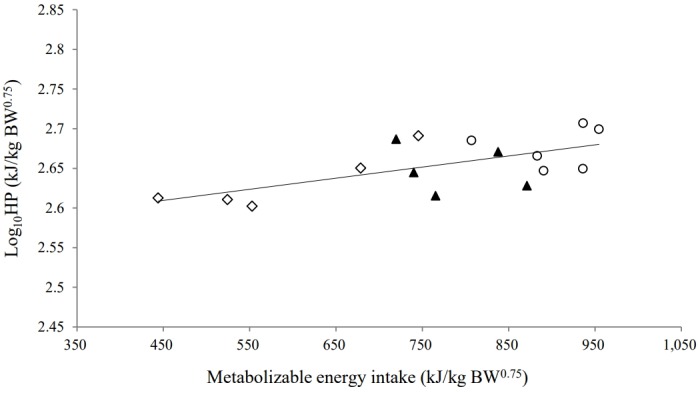
Linear regression of the logarithm of heat production (Log_10_HP, kJ/kg BW^0.75^) against metabolizable energy intake (MEI, kJ/kg BW^0.75^) of yearling Thai native beef cattle fed fermented total mixed ration diets with different proportions [100 (⋄), 300 (▲), and 500 (○) g/kg dry matter basis] of cassava pulp instead of rice straw. Equation: Log_10_HP = (0.00015_(SE=0.00004)_×MEI)–2.53_(SE=0.03)_ (*r* ^2^ = 0.51; p<0.001; residual standard deviation = 0.007).

Yearling Thai native beef cattle fed on the experimental diets exhibited a positive energy balance (energy retention >0). There was a strong correlation between ER and MEI (Figure 1). The ME_m_ requirement of yearling Thai native beef cattle, as derived from the regression equation, was 399 kJ/kg BW^0.75^. In addition, the efficiency of ME utilization above the estimated maintenance for growth (*k*_g_) was 0.86. The correlation between HP and MEI could be expressed as Log_10_HP = 0.00015MEI+ 2.53 (*r* ^2^ = 0.51, n = 16, p<0.001). The fasting HP, or NE_m_, was 338.8 kJ/kg BW^0.75^, estimated as the antilog of the intercept of the linear regression (Figure 2). The ME_m_ (kJ/kg BW^0.75^) was calculated as 388 kJ/kg BW^0.75^ by iteration of the semi-log linear regression equation until HP was equal to MEI. The partial efficiency of ME utilization for maintenance (*k*_m_) was computed as 0.87 by taking the NE_m_ requirement divided by the ME_m_ requirement.

## DISCUSSION

The analyzed chemical composition, energy content and fermentation qualities of rice straw, cassava pulp and other feed ingredients are shown in Table 1. The FTMR diets were formulated to be iso-nitrogenous and EE contents, with values ranging from 96.8 to 99.2 and 58.5 to 58.9 g/kg DM, respectively. Decreases in the NDF, ADF, and ADL contents were observed as the proportion of rice straw in the diet decreased. The FTMR energy density increased as the proportion of cassava pulp in the diet increased; this finding was expected because cassava pulp has a higher NFC content than rice straw.

In this study, 350-kg big-bag silo storage of FTMRs produced good-quality silage that effectively maintained both nutritive and economic values and remained well preserved for more than four weeks. The FTMR fermentation qualities were characterized by low pH, VFA, and NH_3_-N values, and high lactic acid content within seven days of ensiling. These characteristics are consistent with the results of Wang and Nishino [31], who suggested that well-preserved silage should have a pH of 3.7 to 4.2, a high concentration of lactic acid, small quantities of fermentation acids (such as acetic acid, propionic acid and butyric acid) and concentrations of NH_3_-N below 100 g/kg of total N; the presence of NH_3_-N indicates that some deamination of amino acids has occurred during fermentation. We used an ensilage period of at least seven days based on a previous *in vitro* study [17] that indicated that seven days of ensilage resulted in good silage quality (pH 3.8 to 4.0, lactic acid 63 to 66 g/kg DM, butyric acid 0 to 0.03 g/kg DM and NH_3_-N 84 to 97 g/kg total N) and an aerobic stability test indicating no self-heating within 30 h after opening. In our previous study, the three FTMRs before ensiling had a pH of 4.8 to 5.0 and DM, GE, OM, CP, NDF, ADF, and NFC contents of 359 to 366 g/kg, 17.7 to 17.8 MJ/kg DM, 896 to 945 g/kg DM, 96 to 97 g/kg DM, 458 to 647 g/kg DM, 315 to 490 g/kg DM and 113 to 350 g/kg DM, respectively [17].

Based on the OM, NDF, and NFC composition of cassava pulp (Table 1), it was expected that increasing the proportion of cassava pulp in the diet, replacing rice straw, would supply more digestible nutrients and energy because of the higher NFC composition of the diets and would thus improve digestible nutrient intake and growth performance. Our data indicated that the voluntary feed intake of Thai native cattle fed an FTMR diet based on tropical feedstuffs ranged from 2.7 to 3.0 kg/d or a limited DM intake of up to 2.2% of BW (71.5 g/kg BW^0.75^). These feed intake levels may reach the maximum capacity of rumen gut fill and distention. The decreased intake of NDF and ADF by cattle fed cassava pulp at 500 g/kg DM might have been due to the lower NDF and ADF contents of this treatment compared with the 100 and 300 g/kg DM cassava-pulp treatments.

In this study, increasing the proportion of cassava pulp in the diet by replacing rice straw provided sufficient digestible nutrient and energy supply to support average daily growth gains of 391 to 569 g/d. The diets containing 9.6 to 12.4 MJ ME/kg DM and CP (approximately 10%) were formulated to meet or exceed the nutrient requirements of Thai native cattle weighing 100 kg with a target average daily weight gain of 500 g/d. WTSR [4] suggests that the ME and CP intake requirement of Thai native cattle with BW 100 kg and average daily gain 500 g/d is 31 MJ/d and 349 g/d, respectively. In this study, the CP intake was insufficient according to the WTSR [4] recommendation, but average daily gain was 494 and 569 g/d for cattle fed CSP-300 and CSP-500 diets, respectively. The increased growth performance of cattle fed cassava pulp at 500 g/kg DM in the diet was associated with increased digestibility of DM, OM, NDF, and NFC, indicating that cassava pulp contains high levels of digestible carbohydrates that are more readily degraded in the rumen than are the carbohydrates in rice straw, which is rich in lignin and silica (Table 1). Our results are consistent with those reported by Rabelo et al [32], who suggested that increasing the energy density of a diet by increasing the NFC content provides benefits such as increased feed intake, feed digestibility, energy intake and energy balance of cows. One explanation for our results is that the diet containing 500 g/kg DM of cassava pulp provided greater digestibility of NDF and NFC and thus likely promoted changes in the activity of rumen microorganisms, particularly fibrolytic and non-fibrolytic microbes, resulting in the synergistic degradation of fiber. Koike et al [33] demonstrated positive interactions between fibrolytic rumen bacteria (*F. succinogenes*, *R. albus*, and *R. flavefaciens*) and non-fibrolytic bacteria, such as *Selenomonas ruminantium* and *Streptococcus bovis*. The real-time PCR results showed lower population levels of the three main fibrolytic bacteria (Table 3) in the cattle fed 500 g/kg DM cassava pulp. This finding may be due to the decreased fiber substrate and increased population of total protozoa, which can engulf fibrolytic bacteria. Increased cassava pulp in the diet may also promote the growth of other bacterial groups, such as starch-utilizing bacteria or other NFC-utilizing bacteria. This rumen microbial population change should affect the increase in digestible nutrients, energy intake and retention and, thus, improve growth performance. Newbold et al [34] showed evidence suggesting that the degradation of fiber in the rumen requires the synergistic work of several microbes, including fibrolytic bacteria, protozoa and fungi. Additionally, the smaller particle size of cassava pulp (approximately 2 to 3 mm in length) compared with rice straw (approximately 6 to 7 cm in length) might increase nutrient digestibility by increasing surface area, thereby increasing the accessibility of materials to digestive enzymes. Similar results were reported by Miron et al [35], who found that replacing forage with soy hulls, which are a readily digestible crop by-product, resulted in increased feed intake, nutrient digestibility and energy retention in lactating cows.

The energy partitioning results of this study demonstrated that the energy intake, retention and efficiency of yearling Thai native cattle were significantly improved by increasing the proportion of cassava pulp in FTMR-based diets. Our findings confirm previous reports on energy consumption and energy efficiency in tropical zebu beef cattle fed low-quality diets [5,6]. The greatest energy retention was observed in cattle fed a diet containing 500 g/kg DM cassava pulp (Table 3) because of improved digestibility, which decreased the energy lost through feces and urine. The decreases in energy loss from feces and urine were positively correlated with increases in DE and ME intake because the energy content increased as the proportion of cassava pulp in the diet increased. In this study, the replacement of rice straw by cassava pulp caused significant changes in the NDF and ADF fiber content and, consequently the NFC dietary content, with few changes in protein. However, making sufficient GE available in the rumen resulted in a linear decrease in urine and feces energy excretion thus, significantly improving energy retention. Synchronizing the rate of degradation of dietary energy and protein release in the rumen, and their consequences, may also play an important role in improving ruminal fermentation, microbial protein synthesis and nutrient digestibility, microbial protein synthesis and metabolizable protein available to the host animal. Sommart et al [8] demonstrated that rumen microbial biomass, net ^15^N-isotop incorporation into cells and VFA production increased linearly with increasing levels of cassava (15% to 45%) in beef cattle diets. In addition, cassava is a good source of tannins and condensed tannins because phenolic plant secondary compounds readily form a complex with binding proteins that affect rumen microbial population manipulation, digestion, and nutrient and energy utilization. Pathak et al [36] has recently demonstrated that increasing the tannin level significantly improved nitrogen metabolism and growth performance and reduced enteric methane emission by lambs.

The lower fiber contents of the 300 and 500 g/kg DM cassava-pulp diets might have led to reduced manure excretion, consistent with the results of Hales et al [37], in which decreased alfalfa levels in the diet resulted in increased energy intake and retention and decreased energy loss via feces and methane. Our results suggest an opportunity for strategic feeding using cassava starch agro-industrial by-products to improve nutrient and energy supplies for beef cattle in tropical countries.

In this study, daily heat production and enteric methane emissions were similar among the dietary treatments. We obtained smaller values for heat production and enteric methane emissions than those obtained in previous studies on older Thai native cattle [6,13,14]. This difference might reflect differences in the energy costs of the maintenance and production of cattle of differing physiological status and productivity [38]. The ME-to-DE ratio observed in the current study ranged from 0.87 to 0.92 (Table 3), which is within the range of 0.82 to 0.90 reported for Thai cattle fed tropical-based diets [5,13,14].

The analysis of the pooled data in this study resulted in an ME_m_ of 399 kJ/kg BW^0.75^ for yearling Thai native beef cattle (Figure 1). This value is less than the value of 484 kJ/kg BW^0.75^ currently recommended by the WTSR [4] based on a meta-analysis of data from 6 studies involving 18 observations of mature male Thai native cattle, and it is lower than the values suggested for *Bos taurus* [11,12]. Our values were also lower than the range of previously published ME_m_ values (458 to 541 kJ/kg BW^0.75^) reported for growing calves, steers, and mature male Thai native and Brahman cattle [5,6,13,14].

The lower ME_m_ value recorded in our study is likely related to the larger proportional contribution of female cattle in our study relative to previous studies, because sex influences overall physiological status, physical activity and allocation to visceral organs. Some experiments [39] have shown differences in chemical body composition between genders and that the relative size of the internal organs, such as the mass of the liver, is greater for male calves than for female calves. This difference in tissue weight might be related to differences in maintenance energy expenditure [1]. Ferrell and Oltjen [40] reported that variations in body mass, liver mass and mass of the gastrointestinal tract have major impacts on energy expenditure. Ferrell and Jenkins [38] estimated that the ME_m_ for Simmental bulls was 16.5% greater than that for Simmental heifers. However, the studies published to date provide limited data on the ME_m_ requirements of female Thai native beef cattle or Thai Brahman cattle. Therefore, further studies are needed to increase the accuracy of maintenance energy requirements for heifer beef cattle in the tropics.

The *k*_g_ regression analysis yielded a value of 0.86, which is greater than the values of 0.35 and 0.53 previously reported for older mature stage Thai native cattle fed low-quality diets [6,14]. However, our value is similar to the value of 0.86 reported by Kirkland and Gordon [41]. Study differences in *k*_g_ might be due to differences in the age, physiological state, production level and nutrient utilization of the studied cattle, which are influenced by the fat and protein content of tissues and by dietary ME content.

## CONCLUSION

Replacing rice straw with cassava pulp at concentrations up to 500 g/kg of DM in the diet of beef cattle improved growth performance, energy intake and energy retention because of the increase in digestible feed intake and the digestibility of DM, OM, NDF, and NFC. Real-time PCR analysis clearly showed that the populations of total protozoa increased and those of fibrolytic bacteria decreased as the proportion of cassava pulp in the diet increased.

## Figures and Tables

**Table 1:** Analyzed chemical composition of rice straw, cassava pulp and other feed ingredients; chemical composition, energy content, fermentation quality and feed price of the three fermented total mixed ration dietary treatments.

Item	Rice straw	Cassava pulp	Levels of cassava pulp in diet (g/kg DM)		

			100	300	500
Ingredients (g/kg DM)					
Rice straw	-	-	500	300	100
Cassava starch pulp	-	-	100	300	500
Palm kernel meal	-	-	235	235	235
Soybean meal	-	-	50	50	50
Rice bran	-	-	100	100	100
Urea	-	-	5	5	5
Mineral1)	-	-	5	5	5
Premix2)	-	-	5	5	5
Chemical composition (g/kg DM)					
Dry matter (g/kg as fed)	928	238	366	362	364
Organic matter	880	982	895	921	945
Crude protein	27	22	99	97	97
Ether extract	9	8	59	59	59
Neutral detergent fiber	770	328	632	536	452
Acid detergent fiber	516	200	491	391	308
Acid detergent lignin	149	30	83	75	63
Non-fiber carbohydrate3)	74	624	105	229	337
Energy content (MJ/kg DM)					
Gross energy	16.2	16.4	17.3	17.6	17.7
Digestible energy	-	-	11.1	12.6	13.5
Metabolizable energy	-	-	9.6	11.4	12.4
Fermentation quality (g/kg DM)					
pH	-	-	4.0	3.9	3.8
Lactic acid	-	-	64	68	63
Acetic acid	-	-	10	8	7
Propionic acid	-	-	0.54	0.43	0.38
Butyric acid	-	-	0.02	0.00	0.01
Ammonia-N (g/kg total N)	-	-	98	94	92
Feed price					
USD/tonne as-fed	77	14	72	71	69
USD/tonne DM	83	58	198	195	189

DM, dry matter.

1) Minerals included 164 g of Ca, 20 g of P, 11 g of Mn, 132.6 g of Na, 19.2 g of S, 2 g of Fe, 1 g of Cu, 2.888 g of Mg, 0.042 g of Co, 3.582 g of Zn, 0.035 g of I, and 0.027 g of Se (Mineral #0106410029, the Dairy Co–Operatives Federation Thai–Dansk Ltd., Saraburi, Thailand).

2) Vitamin mineral premix containing 2,000 IU/g of vitamin A, 400 IU/g of vitamin D_3_, 3 IU/g of vitamin E, 0.06 g of Se, 1.6 g of Cu, 6 g of Zn, 8 g of Mn, 10 g of Fe, 0.02 g of Co, 10 g of Mg, and 0.10 g of I (T.S. Dairy Mix #0104490710, Thai Serve Co. Ltd., Samut Prakan, Thailand).

3) Calculated according to the formula 1,000–(crude protein+neutral detergent fiber+ether extract+ash).

**Table 2:** Effects of replacing rice straw with cassava pulp in a fermented total mixed ration on growth performance, feed intake and digestibility of Thai native beef cattle (n = 18)

Item	Levels of cassava pulp in diet (g/kg DM)			SEM	Contrast1)	
	
	100	300	500		L	Q
Growth performance						
Initial weight (kg)	101	98	97	2.3	0.58	0.83
Final weight (kg)	163	173	182	9.9	0.20	0.85
Live weight gain (kg)	60	76	85	6.8	0.08	0.72
Average daily gain (g/d)	391	494	569	47.1	<0.05	0.81
Dry matter intake						
kg/d	2.74	2.75	3.01	0.203	0.62	0.79
% of BW	1.85	2.05	2.18	0.061	0.07	0.14
g/kg BW^0.75^	63.9	69.7	71.5	2.54	0.15	0.40
Nutrient intake (kg/d)						
OM	2.45	2.53	2.86	0.185	0.31	0.76
CP	0.27	0.26	0.28	0.021	0.89	0.81
EE	0.19	0.18	0.21	0.014	0.65	0.23
NDF	1.68	1.49	1.36	0.121	0.06	0.99
ADF	1.29	1.06	0.84	0.095	<0.05	0.88
NFC	0.38	0.61	1.09	0.095	<0.01	0.28
Digestible DM	1.55	1.86	2.26	0.143	<0.05	0.95
Digestible OM	1.50	1.80	2.22	0.139	<0.05	0.83
Digestible CP	0.15	0.18	0.18	0.011	0.22	0.28
Digestible NDF	0.87	0.89	0.88	0.099	0.77	0.74
Digestible NFC	0.32	0.55	1.03	0.119	<0.01	0.68
Digestibility (%)						
DM	56.4	68.1	74.7	1.72	<0.01	0.24
OM	60.9	71.2	77.5	1.52	<0.01	0.29
CP	60.5	63.7	60.6	1.91	0.99	0.20
EE	91.2	94.2	90.0	1.30	0.42	0.39
NDF	51.9	59.6	64.7	2.01	<0.01	0.63
ADF	49.6	50.5	51.9	2.88	0.64	0.87
NFC	84.0	88.8	95.5	2.25	<0.01	0.73

DM, dry matter; SEM, standard error of the mean; BW, body weight; OM, organic matter; CP, crude protein; EE, ether extract; NDF, neutral detergent fiber; ADF, acid detergent fiber; NFC, non-fiber carbohydrate.

1) Probability value of orthogonal polynomial contrast; L, linear; Q, quadratic.

**Table 3:** Effects of replacing rice straw with cassava pulp in the fermented total mixed ration on rumen pH; microbial populations; and energy partitioning, intake and efficiency by Thai native beef cattle (n = 18)

Item	Levels of cassava pulp in diet (g/kg DM)			SEM	Contrast1)	
	
	100	300	500		L	Q
Ruminal pH	7.0	6.7	6.8	0.12	0.20	0.26
Ruminal microbial population2)						
Total protozoa (×10^9^)	0.2	0.5	1.7	0.04	<0.05	0.35
Total bacteria (×10^12^)	8.9	7.6	6.6	1.24	0.21	0.91
*Fibrobacter succinogenes* (×10^9^)	3.7	1.5	1.7	0.06	<0.05	0.14
*Ruminococcus flavefaciens* (×10^10^)	1.9	1.4	0.2	0.04	<0.01	0.43
*Ruminococcus albus* (×10^9^)	1.5	0.8	0.2	0.03	<0.05	0.85
Total methanogens (×10^9^)	1.4	1.2	1.6	0.23	0.55	0.41
Rumen cluster C archaea (×10^8^)	1.0	0.2	0.2	0.03	0.07	0.32
Energy partition (MJ/d)						
Gross energy intake	47.4	48.9	53.4	3.42	0.50	0.94
Feces excretion	16.9	14.2	12.8	1.58	0.07	0.91
Urine excretion	0.7	0.5	0.4	0.048	<0.01	0.33
Methane emission	3.2	2.8	2.8	0.28	0.30	0.58
Heat production	18.4	17.6	18.4	1.30	0.99	0.63
Energy retention	8.2	13.8	17.3	2.19	<0.05	0.62
Energy intake (kJ/kg BW^0.75^)						
Gross energy (GE)	1,106	1,238	1,266	39.0	<0.05	0.19
Digestible energy (DE)	708	880	963	44.8	<0.01	0.35
Metabolizable energy (ME)	616	796	886	43.8	<0.01	0.31
Energy efficiency						
DE/GE	0.64	0.71	0.76	0.029	<0.05	0.73
ME/GE	0.56	0.64	0.70	0.030	<0.05	0.60
ME/DE	0.87	0.90	0.92	0.007	<0.05	0.12

SEM, standard error of the mean.

1) Probability value of orthogonal polynomial contrast: L, linear; Q, quadratic.

2) DNA copies/mL of rumen fluid.
